# Three-year assessment of cadmium exposure and bone mineral density changes in cadmium-contaminated areas in northwestern Thailand

**DOI:** 10.1371/journal.pone.0334521

**Published:** 2025-10-22

**Authors:** Aroon La-up, Udomsak Saengow, Tawisa Umpong, Phuwasin Buakate, Mondhakarn Oprasertsawat

**Affiliations:** 1 Mahidol University, Nakhonsawan Campus, Nakhonsawan, Thailand; 2 Center of Excellence in Data Science for Health Study, Walailak University, Thai Buri, Tha Sala, Nakhon Si Thammarat, Thailand; 3 School of Medicine, Walailak University, Thai Buri, Tha Sala, Nakhon Si Thammarat, Thailand; 4 Research and Innovation Institute of Excellence, Walailak University, Thai Buri, Tha Sala, Nakhon Si Thammarat, Thailand; 5 Mae Sot General Hospital, Mae Sot District, Tak Province, Thailand; 6 School of Public Health, Walailak University, Thai Buri, Tha Sala, Nakhon Si Thammarat, Thailand; Makerere University College of Natural Sciences, UGANDA

## Abstract

Chronic cadmium exposure is linked to bone loss, but its effect on the short-term progression of bone mineral density (BMD) in previously exposed populations is not well understood. This study aimed to investigate the association between baseline urinary cadmium (U-Cd) levels and the subsequent three-year change in BMD in a chronically exposed cohort in northwestern Thailand. We conducted a three-year longitudinal study of 393 residents (40 men, 353 women) aged 35 and older. Baseline (2019) U-Cd was the primary exposure, and BMD at the calcaneus was measured in 2019 and 2022. Dual-energy X-ray absorptiometry was used for BMD measurements and atomic absorption spectrometry for U-Cd analysis. The mean U-Cd levels in males (7.18 ± 1.35 μg/g creatinine) were significantly higher (p < 0.001) than in females (3.68 ± 2.13 μg/g creatinine). This study found a statistically significant decrease in BMD in the group with the lowest U-Cd levels (<2.0 μg/g creatinine, p = 0.001) and in the overall sample (from 0.392 ± 0.079 μg/g creatinine in 2019 to 0.384 ± 0.094 μg/g creatinine in 2022, p = 0.004). However, no statistically significant changes were observed in groups with U-Cd levels above 2.0 μg/g creatinine. Osteoporosis prevalence remained stable in both males and females. The Linear Mixed-Effects Model analysis revealed significant associations between BMD and several factors: increasing age, female sex, diabetes status and BMI. Age and female sex were negatively associated with BMD, while BMI showed a positive relationship. U-Cd levels were not significantly associated with BMD changes over the three-year period (coefficient = −0.002, p = 0.073), though a slight downward trend in BMD was observed across all cadmium exposure levels. This study underscores the complexity of cadmium’s effects on bone health and emphasizes the need for longer-term follow-up studies to better understand the potential cumulative impact of cadmium exposure on BMD.

## Introduction

Heavy metals, also known as potentially toxic elements, are widespread environmental pollutants with long biological half-lives and adverse health effects. They can accumulate in various tissues and have been linked to kidney, neurological, cardiovascular, and skeletal disorders [1]. Cadmium exposure poses a persistent threat to bone health, with implications that can span decades. Environmental exposure to cadmium remains a significant public health concern worldwide, particularly in areas with industrial contamination or extensive agricultural practices. Cadmium, a toxic heavy metal, has been associated with a wide range of adverse health effects, such as renal dysfunction, skeletal disorders, cardiovascular disease, neurodevelopmental impairment, and carcinogenicity [2,3]. The impact of cadmium on bone health has garnered increasing attention due to its potential to induce osteoporosis and increase fracture risk, even at relatively low exposure levels [4].

Osteoporosis is a major global health concern, especially among the elderly population. A previous study found that the global prevalence of osteoporosis in older adults is approximately 18.3%, with variations across different regions [5], highlighting the importance of environmental and lifestyle factors that may affect bone health. The relationship between cadmium and osteoporosis has been confirmed by several studies [6,7], which have found that long-term exposure to low levels of cadmium can lead to bone mass loss and increase the risk of osteoporosis. Previous studies have reinforced that chronic low-level cadmium exposure can interfere with calcium metabolism and bone formation processes, leading to decreased bone mineral density (BMD) and increased bone fragility [8]. The mechanism by which cadmium affects bone metabolism is multifaceted. Current research suggests it involves direct effects on osteoblast and osteoclast function, as well as indirect effects through disruption of calcium homeostasis and hormonal regulation [9]. The cumulative nature of cadmium exposure suggests that its effects on bone health may progressively worsen over time, emphasizing the need for longer-term studies to elucidate these patterns.

Cadmium contamination in northwestern Thailand has been extensively documented since the early 2000s. Previous investigations have reported elevated levels of Cd and Zn in paddy soils and rice grains in agricultural communities located downstream of zinc-mineralized zones influenced by upstream mining activities, leading to chronic dietary exposure among residents who consume locally grown rice [10,11]. Our previous study conducted in 2019 provided valuable insights into the long-term effects of cadmium exposure on bone health in a contaminated area in northwestern Thailand. Conducted 15 years after measures were implemented to reduce cadmium exposure, this research revealed critical findings about the persistent impact of cadmium on skeletal health. Despite decreased environmental cadmium levels, the prevalence of osteoporosis remained significantly higher in the exposed population compared to those in non-contaminated areas. This discovery underscores the enduring nature of cadmium’s effects on bone health and emphasizes the crucial need for ongoing monitoring and intervention in affected communities [12,13].

While previous research has established the long-term effects of cadmium on bone health, questions remain about the progression of these effects in populations with continuous exposure. Building upon these findings, this study aims to determine the association between baseline urinary cadmium (U-Cd) levels and the subsequent three-year rate of change in BMD in this chronically exposed cohort. This longitudinal approach provides a more nuanced understanding of how baseline cadmium exposure influences the progression of bone health deterioration of bone health in already impacted populations. By examining these changes, we aim to identify potentially indicators of accelerated BMD reduction, which could inform more timely and targeted public health interventions in populations living in cadmium-contaminated areas.

## Methodology

### Ethical approval

This study was reviewed and approved by the Ethics Committee in Human Research, Walailak University (Approval No. WUEC-22-178-01). All participants provided written informed consent before their participation in the study.

### Study design and population

This three-year longitudinal study is a follow-up of the cohort from our 2019 investigation [12]. The baseline cohort in 2019 was recruited from residents living in the cadmium-contaminated areas of Mae Sot District, northwestern Thailand, where contamination was first reported in 2004. The inclusion criteria for the 2019 study were residents aged 35 years and older who had been living in the cadmium-contaminated areas since at least 2004. The original publication did not specify any exclusion criteria.

Of the original 934 participants from the 2019 study, 393 individuals were successfully followed up in 2022. This analysis includes only the 393 participants who had complete data at both survey waves. This method enables us to examine changes in BMD and its association with U-Cd levels at the individual level over the three-year period.

The study population consisted of residents aged 35 years and older who had been living in the cadmium-contaminated areas since at least 2004, and who participated in our previous study. This continuity in the study population enabled us to assess the ongoing effects of cadmium exposure on bone health, even after the implementation of exposure reduction measures.

By utilizing a repeated measures design and following up on our previous cohort, we could directly capture changes in BMD and its association with cadmium exposure within the same individuals over the three-year period. This approach provides valuable insights into the evolving patterns of cadmium-related BMD reduction and allows for a more comprehensive understanding of the long-term impacts of cadmium exposure on bone health.

### Data collection

Participants completed a comprehensive questionnaire at both time points (2019 and 2022) to collect detailed demographic and health information. The questionnaire included items regarding personal characteristics (age, sex,), lifestyle factors (smoking status, alcohol consumption, and exercise habits), and anthropometric measurements (body weight, height, waist circumference).

### Urinary cadmium measurement

We used the baseline U-Cd data from our 2019 investigation for this analysis. Given cadmium’s long biological half-life in the human body (10–30 years) [14] these measurements are considered valid indicators of chronic exposure and body burden at the time of follow-up in 2022.

In 2019, second-voided morning urine samples were collected from each participant using cadmium-free plastic bottles. A 3-mL aliquot from each sample was prepared and stored at −20°C until analysis. U-Cd concentrations were determined using graphite furnace atomic absorption spectrometry (GF-AAS; Varian Model AA280Z, Palo Alto, CA) at the laboratory of Mae Sot General Hospital, which is accredited by the Thailand Medical Technology Council. The analysis was conducted at a wavelength of 228.8 nm, and calibration was performed using certified standards from the National Institute of Standards and Technology (NIST, USA). Quality assurance and control were performed using Lyphocheck® reference materials (Bio-Rad, Gladesville, New South Wales, Australia). These procedures were consistent with those described in our previous peer-reviewed publications [12,15,16], ensuring methodological continuity in terms of sample collection, storage, and laboratory analysis. We categorized the U-Cd levels into four groups: < 2, 2–4.9, 5–9.9, and ≥10 μg/g creatinine. This categorization allows for the assessment of dose-response relationships and maintains consistency with our previous studies, facilitating comparisons across time. By using these previously measured U-Cd levels, we can examine how the initial cadmium body burden relates to changes in BMD over the three-year follow-up period, providing insights into the long-term effects of cadmium exposure on bone health.

### Bone mineral density assessment

BMD was assessed at the calcaneus using dual-energy X-ray absorptiometry (DXA) with the EXA-3000 device (Osteosys, Seoul, South Korea), consistent with our previous research protocol. The same machine was used in both 2019 and 2022. The BMD measurements were performed by a technician experienced in operating the device, and calibration procedures were performed regularly to ensure consistency across time points. Osteoporosis was defined according to World Health Organization (1994) [17] criteria as a T-score ≤ −2.5, based on reference data from healthy adults aged 20–50 years.

### Statistical analysis

Data management and analysis were performed using R version 4.0.2, with relevant packages including `lme4` (v1.1-23) and `ggplot2` (v3.3.2). The analysis focused on evaluating changes in bone mineral density (BMD) and its association with urinary cadmium (U-Cd) over a three-year period.

Descriptive statistics (means, standard deviations, frequencies, percentages) were used to summarize participant characteristics. Normality of continuous variables was assessed using Shapiro-Wilk tests and Q-Q plots. Based on distributional properties, changes between 2019 and 2022 were assessed using either paired t-tests or Wilcoxon signed-rank tests. Categorical comparisons employed Fisher’s exact test.

Outliers were screened using standardized residuals, and no influential points were excluded. All participants had complete data for the variables of interest, and no imputation or exclusion due to missingness was required. A significance threshold of 0.05 was used throughout. To evaluate the effect of U-Cd on BMD changes, linear mixed-effects models were constructed using the `lme4` package. The models included fixed effects for U-Cd (continuous), time (2019 vs 2022), their interaction, and covariates (age, sex, BMI, smoking status). Participant ID was included as a random effect to account for individual variations, allowing us to estimate the effects of these variables on BMD and their statistical significance.

To investigate the potential for a non-linear relationship between urinary cadmium and bone mineral density, we fitted a linear mixed-effects model incorporating restricted cubic splines with four knots for the urinary cadmium variable. This non-linear model was formally compared to the primary linear model using a likelihood ratio test to assess the assumption of linearity. Additionally, to address potential sex-specific effects, we conducted a stratified analysis by fitting the primary linear mixed-effects model separately for male and female subgroups.

## Results

Table 1 shows the demographic characteristics and clinical variables of the study participants (males, *n* = 40, females, *n* = 353) in 2019 and 2022. The mean age increased significantly in both sexes (p < 0.001), while the body mass index (BMI) remained stable. Waist circumference in women increased slightly (p = 0.041). BMD did not change significantly. Smoking behavior, alcohol consumption, and exercise habits did not differ significantly between the two time points. Regarding U-Cd levels, this study used only the 2019 measurements for both time points. This decision was based on the extremely long biological half-life of cadmium in the human body, estimated to be between 10–30 years. Given this prolonged half-life, the 2019 U-Cd levels are considered representative of the participants’ long-term cadmium exposure and body burden, with no significant changes expected over the three-year study period. In men, the mean U-Cd level was 7.18 ± 1.35 μg/g creatinine, and in women, it was 3.68 ± 2.13 μg/g creatinine. A statistically significant difference in U-Cd levels was found between males and females (p < 0.001), with males having higher U-Cd levels than females.

**Table 1:** Characteristics of the study participants for continuous variables.

Variables	Male (n = 40)			Female (n = 353)		
	2019	2022	p-value	2019	2022	p-value
Age (year)	61.6 ± 9.1	64.3 ± 9.1	0.176^a^	58.3 ± 7.1	61.3 ± 7.0	<0.001^a^
Age group (year)						
35–44	2 (5.0)	1 (2.5)	0.004^b^	5 (1.4)	2 (0.6)	0.005^b^
45–54	7 (17.5)	6 (15.0)		108 (30.6)	49 (13.9)	
55–44	16 (40.0)	13 (32.5)		181 (51.3)	205 (58.1)	
≥ 65	15 (37.5)	20 (50.0)		59 (16.7)	97 (27.5)	
BMI (kg/m²)	22.3 ± 3.3	22.0 ± 3.0	0.667^a^	24.5 ± 3.9	24.5 ± 4.3	0.973^a^
Waist (cm.)	82.4 ± 9.1	82.2 ± 9.7	0.916^a^	81.0 ± 13.7	82.1 ± 10.2	0.041^a^
Smoke						
Never	11 (27.5)	11 (27.5)	0.959^b^	303 (85.8)	300 (85.0)	0.664^b^
Ex-smoked	13 (32.5)	15 (37.5)		29 (8.2)	35 (9.9)	
Current	16 (40.0)	14 (35.0)		21 (5.9)	18 (5.1)	
Alcohol						
Never	5 (12.5)	5 (12.5)	0.662^b^	218 (61.8)	204 (57.8)	0.068^b^
Former	10 (25.0)	14 (35.0)		42 (11.9)	64 (18.1)	
Occasionally	24 (60.0)	19 (47.5)		93 (25.3)	85 (21.4)	
Every day	1 92.5)	2 (5.0)		–	–	
Exercise						
0 days/week	21 (52.5)	22 (55.0)	0.775^b^	172 (48.7)	178 (50.4)	0.087^b^
< 3 days/week	14 (35.0)	11 (27.5)		112 (31.7)	88 (24.9)	
3 days/week	4 (10.0)	4 (10.0)		21 (5.9)	18 (5.1)	
4–6 days/week	–	–		9 (2.5)	19 (5.4)	
7 days/week	1 (2.5)	3 (7.5)		39 (11.0)	50 (14.2)	
Urinary cadmium (μg/g creatinine)						
< 2	–	–		80 (22.7)	80 (22.7)	1.00^b^
2.0–4.9	–	–		120 (34.0)	120 (34.0)	
5.0–9.9	35 (87.5)	35 (87.5)	1.00^b^	130 (36.8)	130 (36.8)	
* ≥ *10	5 (12.5)	5 (12.5)		23 (6.5)	23 (6.5)	
mean ± SD	7.18 ± 1.35			3.68 ± 2.13		<0.001^a^
BMD	0.47 ± 0.08	0.48 ± 0.10	0.475^a^	0.38 ± 0.07	0.37 ± 0.09	0.083^a^

Values are presented as mean ± standard deviation (SD) for continuous variables and n (%) for categorical variables.

^a^independent samples t-tests

^b^Fisher’s exact test for comparing 2019 vs 2022 within each sex group

Table 2 shows analysis of BMD differences between 2019 and 2022, stratified by U-Cd levels. Statistically significant decreases in BMD were observed in the group with U-Cd levels <2 μg/g creatinine (mean difference −0.015, p = 0.001) and in the overall sample (mean difference −0.008, p = 0.004). Subjects with U-Cd levels 2–4.99 μg/g creatinine showed a marginally significant decrease (mean difference −0.010, p = 0.074). No statistically significant changes were found in the 5–9.99 μg/g creatinine group (p = 0.331) and the ≥ 10 μg/g creatinine group (p = 0.834). These findings suggest that during the study period, lower U-Cd levels were associated with more pronounced decreases in BMD. The most significant BMD reduction was observed in the lowest cadmium exposure group.

**Table 2:** The difference of BMD between 2019 and 2022 classify by grop of urinary camium levels measured in 2019.

Urinary cadmium (µg/g creatinine)	N	BMD 2022	BMD 2019	Mean diff	95% CI	p-value
		Mean±SD	Mean±SD			
All	393	0.384 ± 0.094	0.392 ± 0.079	−0.008	−0.013, −0.003	0.004
<2.0	80	0.394 ± 0.074	0.410 ± 0.064	−0.015	−0.025, −0.006	0.001
2.0–4.9	120	0.372 ± 0.081	0.382 ± 0.068	−0.010	−0.020, 0.001	0.074
5.0–9.9	165	0.389 ± 0.106	0.393 ± 0.089	−0.004	−0.013, 0.004	0.331
≥10	28	0.375 ± 0.123	0.378 ± 0.092	−0.003	−0.027, 0.022	0.834

*Results test by paired t-tests.

**Fig 1:**
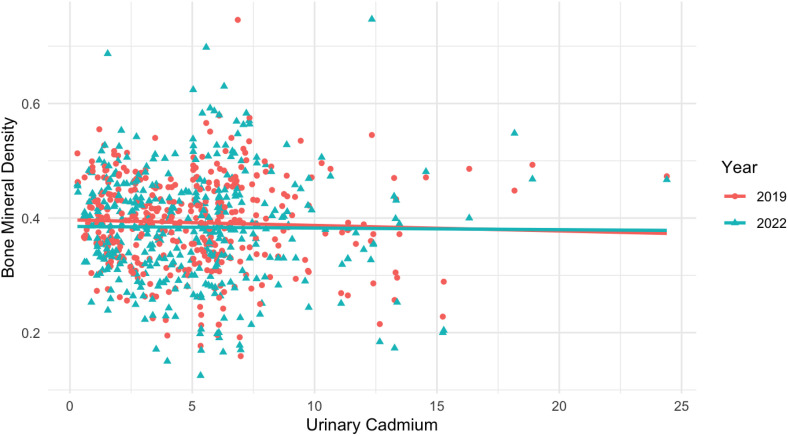
A slight negative trend in the relationship between U-Cd levels and BMD in both 2019 and 2022, as evident from the slightly downward-sloping regression lines in both years. However, no statistically significant relationship was found.

Figure 1 illustrates the relationship between U-Cd levels and BMD in 2019 and 2022. A slight negative trend was observed, as indicated by the slightly downward-sloping regression lines in both years. However, no statistically significant relationship was found.

No statistically significant differences were found in osteoporosis prevalence between 2019 and 2022 for both males (p = 1.00) and females (p = 0.558). However, a slight decrease in prevalence was observed in males, while a slight increase was noted in females across most U-Cd level groups (Table 3).

**Table 3:** Prevalence of osteoporosis classified by sex, and urinary cadmium level compare between 2019 and 2022.

Urinary cadmium (μg/g creatinine)	Male (n = 40)		Female (n = 353)	
	2019	2022	2019	2022
All	16 (40.0)	11 (27.5)	84 (23.8)	100 (28.3)
<2	–	–	18 (5.1)	22 (6.2)
2.0–4.9	–	–	37 (10.5)	39 (11.0)
5.0–9.9	14 (35.0)	10 (25.0)	21 (5.9)	33 (9.3)
≥ 10	2 (5.0)	1 (2.5)	8 (2.3)	6 (1.7)
p-value	1.00		0.558	

*Results test by Fisher’s exact test.

Table 4 presents results from a Linear Mixed-Effects Model analysis of BMD changes over a 3-year period, incorporating interaction terms and adjusting for all covariates. Significant main effects were found for age, female sex, BMI, year, and diabetes status. Specifically, increasing age was associated with a reduction in BMD (coefficient = −0.005 per year, p < 0.001), females had lower BMD than males (coefficient = −0.170, p < 0.001), higher BMI was positively associated with BMD (coefficient = 0.004, p < 0.001), and the presence of diabetes was associated with a slight increase in BMD (coefficient = 0.011, p = 0.030). Additionally, the year 2022 showed a slight increase in BMD compared to 2019 baseline (coefficient = 0.008, p = 0.011). In this fully adjusted model, baseline urinary cadmium did not show a significant main effect on BMD changes (coefficient = −0.004, p = 0.574). Furthermore, the interaction terms revealed that age, sex, and smoking status did not significantly modify the effect of cadmium on BMD.

**Table 4:** Linear Mixed-Effects Model analysis of bone mineral density changes in relation to urinary cadmium levels in a 3-Year Period.

Variables	Coefficient	95% CI	Std. Error	p-value
Urinary Cadmium	−0.004	−0.017, 0.009	0.007	0.574
Age	−0.005	−0.006, −0.003	0.001	<0.001
Sex: Female	−0.168	−0.232, −0.109	0.032	<0.001
Smoke: Former	−0.035	−0.075, 0.003	0.020	0.080
Smoke: Current	−0.018	−0.066, 0.033	0.025	0.475
Year:2022	0.008	0.001, 0.013	0.003	0.011
BMI	0.004	0.003, 0.006	0.001	<0.001
DM: Yes	0.011	0.001, 0.021	0.005	0.030
Urinary Cadmium * Age	−0.00002	−0.0002, 0.0001	0.0001	0.804
Urinary Cadmium * Sex: Female	0.003	−0.004, 0.012	0.004	0.427
Urinary Cadmium * Smoke: Former	0.003	−0.003, 0.010	0.003	0.372
Urinary Cadmium * Smoke: Current	−0.001	−0.009, 0.006	0.004	0.748

**Fig 2:**
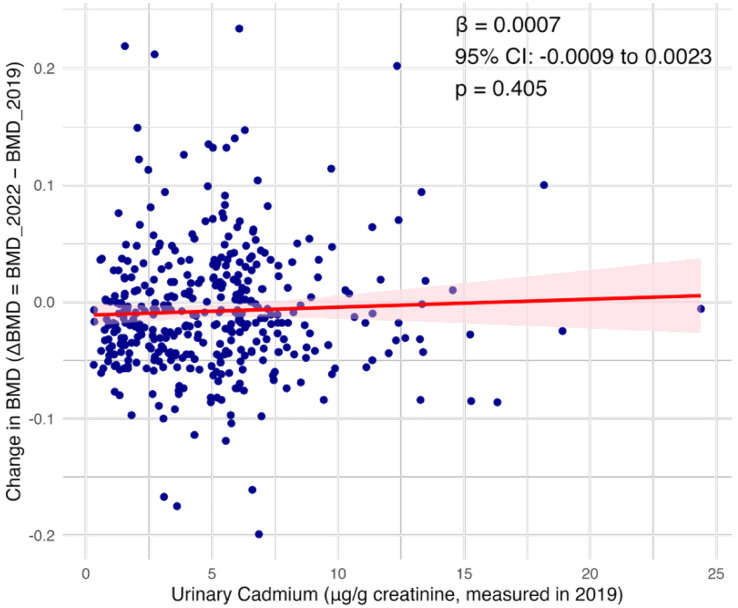
Association between baseline urinary cadmium and the three-year change in bone mineral density (ΔBMD). Each point represents an individual participant. The solid red line shows the linear regression fit, and the shaded area represents the 95% confidence interval. No statistically significant linear association was observed (β = 0.0007, 95% CI: -0.0009 to 0.0023, p = 0.405).

Association between urinary cadmium level (measured in 2019) and the change in bone mineral density (ΔBMD = BMD 2022 – BMD 2019) over a three-year period. The red line represents the linear regression fit with a 95% confidence interval (shaded area). No significant association was observed (β = 0.0007, 95% CI: −0.0009 to 0.0023, p = 0.405), as illustrated in Fig 2.

**Fig 3:**
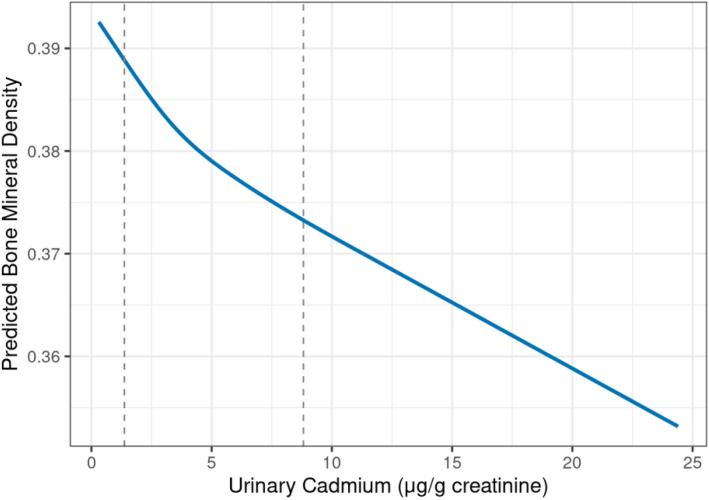
Non-linear relationship between urinary cadmium and bone mineral density from the adjusted restricted cubic splines model. The solid blue line represents the predicted bone mineral density after adjusting for age, sex, smoking status, year, BMI, and diabetes status. The dashed vertical lines indicate the 10th and 90th percentiles of the urinary cadmium distribution. The analysis did not reveal a significant non-linear relationship (p-value for non-linearity = 0.8953).

The analysis using restricted cubic splines did not reveal a significant non-linear relationship between urinary cadmium and bone mineral density (p-value for non-linearity = 0.8953), as illustrated in Fig 3.

In the analysis stratified by sex, no statistically significant association was found between urinary cadmium and bone mineral density in either the male subgroup (coefficient = −0.0037; p = 0.439) or the female subgroup (coefficient = −0.0016; p = 0.083). These results are presented in Table 5.

**Table 5:** Stratified analysis of the association between urinary cadmium and bone mineral density, by sex.

Sex	n	β-coefficient^a^	95% Confidence Interval	P-value
Male	40	−0.004	−0.013, 0.006	0.439
Female	353	−0.002	−0.004, 0.0002	0.083

^a^β-coefficient represents the change in bone mineral density per 1 µg/g creatinine increase in urinary cadmium, derived from a linear mixed-effects model. Models were adjusted for age, smoking status, year, body mass index (BMI), and diabetes mellitus status.

## Discussion

This study examined the relationship between U-Cd and changes in BMD among residents in a cadmium-contaminated area in northwestern Thailand over a three-year period (2019–2022). As a continuation of earlier research reporting osteoporosis status after 15 years of exposure reduction [12], the study employed a longitudinal design with repeated measures on the same cohort to capture short-term BMD changes potentially linked to cadmium exposure.

Our study found significant changes in BMD among the population with varying levels of U-Cd over a 3-year period. Interestingly, the most pronounced and statistically significant decrease in BMD was observed in the group with the lowest U-Cd levels (<2 μg/g creatinine, p = 0.001). This finding contrasts with the traditional understanding that higher cadmium exposure leads to greater bone loss. The overall sample also showed a significant decrease in BMD (p = 0.004), while the group with U-Cd levels of 2–4.99 μg/g creatinine showed a marginally significant decrease (p = 0.074). However, it is crucial to note that groups with higher U-Cd levels (5–9.99 and ≥10 μg/g creatinine) had lower baseline BMD values in 2019 and did not show statistically significant changes in 2022. This observation suggests that individuals with higher cadmium exposure may have already experienced substantial BMD reduction prior to our study, resulting in lower initial BMD levels. The lack of significant change in these groups over the three-year period could indicate a slower rate of BMD decline after prolonged exposure.

In an additional linear regression analysis using the change in BMD per individual (ΔBMD = BMD 2022 – BMD 2019) as the dependent variable and U-Cd levels measured in 2019 as the independent variable, no significant association was found (β = 0.0007, 95% CI: −0.0009 to 0.0023, p = 0.405; see Figure 2). This result supports the main finding from the mixed-effects model and stratified analyses, suggesting that urinary cadmium level is not significantly associated with short-term changes in BMD within the three-year period.

This pattern contrasts with prior studies linking higher cadmium levels to reduced BMD and increased osteoporosis risk. For example, long-term studies in China and South Korea documented significant BMD reductions in populations chronically exposed to cadmium [18,19]. However, those studies generally spanned longer durations (≥10 years), which may be critical to detecting the cumulative skeletal effects of cadmium. In our study, the short three-year window might be insufficient to capture further significant changes in those already heavily exposed. Our investigation into the shape of the relationship, which explored both linear and non-linear models using restricted cubic splines, did not find a statistically significant association between urinary cadmium and bone mineral density in either analysis. The inverse association observed could reflect residual effects in those with prior high exposure, or vulnerability in subgroups with ostensibly lower current exposure but persistent cumulative burden. These dynamics highlight the importance of considering both exposure duration and timing when assessing cadmium’s skeletal effects. Furthermore, while cross-sectional studies often report negative correlations between cadmium and BMD, such associations may not fully capture temporal variations in exposure or individual susceptibility. For instance, research in postmenopausal women has shown that even low-to-moderate cadmium levels can exacerbate bone loss due to estrogen deficiency [20]. In our study, although the prevalence of osteoporosis did not significantly increase in any group over the three years, a slight rise was observed among women, while it decreased among men. Although not statistically significant, this trend aligns with literature indicating heightened skeletal sensitivity in postmenopausal women. The lack of significant BMD change in high U-Cd groups also raises questions about potential threshold or saturation effects. These individuals may have experienced early and severe bone demineralization, with little further measurable loss during our study.

Our previous study in the same population found high cadmium concentrations in locally consumed rice, identifying dietary intake as a key exposure pathway [12]. Although environmental measurements were not collected in the current study, prior research in the same area found elevated cadmium levels in locally consumed cooked rice. That study established a dose–response relationship between cadmium intake from rice and U-Cd levels, confirming rice as a major exposure pathway for residents [15]. These findings support the interpretation that dietary exposure through locally grown rice remains a relevant contributor to cadmium burden in this population.

We also compared our findings to other population-based studies. In contrast to our results, a recent U.S. cross-sectional analysis reported a negative association between blood cadmium and BMD [21]. Similarly, a study among Chinese women linked high cumulative cadmium intake to elevated risks of osteoporosis and fractures [18,22]. South Korean research further demonstrated sex-specific and obesity-modified effects, with stronger associations observed in obese men [23]. These discrepancies may stem from differences in study design, exposure sources, population characteristics, and follow-up durations. One possible explanation for the contrast is that cadmium’s osteotoxic effects accumulate gradually and may require longer follow-up periods to detect. Our study period of three years may simply be too brief to capture these gradual changes. Indeed, the higher-exposure groups already had low BMD at baseline, suggesting prior damage had occurred, consistent with the slow and cumulative nature of cadmium’s impact on bone.

Our study also contributes to the understanding of biological mechanisms. Cadmium is known to disrupt osteoblast and osteoclast function and induce apoptosis in bone tissue over long-term exposure [9,24]. Although our study did not directly assess these molecular endpoints, the observed BMD trends, particularly the mild but consistent decline across all U-Cd groups, may indicate early signs of continued cadmium-related skeletal impairment.

The complexity of these relationships is further underscored by the mixed-effects model results. Age and female sex were both significantly associated with decreased BMD, consistent with known age and sex related risks [25]. Higher BMI was associated with increased BMD, supporting the protective role of greater body mass [26]. Interestingly, our fully adjusted model also revealed that diabetes mellitus (DM) was significantly associated with a slight increase in BMD (β = 0.011, p = 0.030), a finding consistent with some clinical literature reporting higher bone mass in individuals with type 2 diabetes, although this often paradoxically coexists with increased fracture risk due to poorer bone quality [27]. Smoking status did not show significant associations, though coefficients suggested a possible negative trend. These nonsignificant results could reflect sample size limitations or heterogeneity in smoking behaviors. Interestingly, we observed significantly higher U-Cd concentrations in men compared to women (7.18 vs 3.68 µg/g creatinine, p < 0.001). This finding contrasts with typical patterns, where women are generally more susceptible to cadmium accumulation than men. In our population, however, the gender difference may be partly explained by markedly higher smoking prevalence among men, 35% were current smokers compared to only 5.1% of women. Smoking is a well-established source of cadmium exposure, and recent studies, such as Zhang et al. [28], have demonstrated a strong association between smoking status and U-Cd levels. These behavioral differences may therefore account for the unexpectedly elevated U-Cd concentrations observed among male participants. Importantly, the analysis showed no significant interaction between U-Cd and age, sex, or smoking status in predicting BMD changes. Furthermore, in our analysis stratified by sex, no statistically significant association was found in either men or women, although the potential for low statistical power in the male subgroup should be noted. This may suggest that within the three-year timeframe, cadmium’s effects on bone do not differ markedly across these demographic factors, or that the effects are too subtle to detect in the short term. Nonetheless, the consistently negative trend in BMD across all cadmium exposure groups warrants close observation in future studies.

Our study also found no statistically significant differences in osteoporosis prevalence between 2019 and 2022. This persistently high prevalence is consistent with an earlier cross-sectional analysis in the same population by Limpatanachote et al. (2010) [13] who reported an osteoporosis prevalence of 21.5% in women and 14.7% in men based on 2007 data. This consistent with the idea that cadmium’s skeletal effects may require longer durations to manifest clinically. Previous experimental studies in rats have shown no short-term changes in BMD from cadmium exposure, but significant effects emerge over extended periods [29]. Similarly, epidemiological studies show that adverse bone outcomes may only become evident after years of accumulation [19,30].

While the absence of a significant relationship between U-Cd and BMD changes in this three-year period might suggest a limited short-term impact, the downward trend in BMD across all exposure groups supports continued concern about cadmium’s long-term skeletal effects. These findings are consistent with our previous 15-year study, which showed a significant negative correlation between U-Cd and BMD after prolonged exposure [12]. The contrast between the two studies underscores the importance of study duration when evaluating chronic toxicant effects, particularly for compounds like cadmium with long biological half-lives.

Our findings suggest that cadmium’s effects may follow a non-linear dose–response curve, or that prior exposure has led to irreversible skeletal changes in higher-exposure groups. The decline in BMD among low and mid-exposure groups could reflect the latency of cadmium toxicity, whereby damage continues even after exposure reduction. Such a latency effect may explain why the full benefits of environmental interventions are not yet apparent.

Furthermore, our findings advocate for the integration of additional bone health biomarkers in future research. While BMD is the clinical standard, early metabolic changes may precede measurable density loss. Markers such as bone turnover indicators or inflammatory cytokines may provide earlier signals of cadmium-induced bone pathology. The use of a longitudinal design and repeated measures in the same cohort strengthens the internal validity of our findings. This approach enables within-subject comparisons, reducing variability from individual baseline differences. However, the study also has limitations. The relatively short follow-up period and absence of updated environmental cadmium measurements may limit the interpretation of exposure trends. Moreover, we did not measure other potential bone-toxic metals, such as lead or arsenic, which could confound the observed associations. Despite these limitations, this study contributes important evidence on the temporal dynamics of cadmium exposure and BMD. By building on prior work in the same population, we provide insight into the short-term effects following cadmium reduction interventions. While no statistically significant association was observed between U-Cd levels and BMD over three years, the general decline in BMD across all groups particularly among women signals the need for continued public health vigilance.

### Strengths and limitations

This study builds upon earlier work, providing continuity and allowing for comparisons with previous findings. The two-time point study design allows for the assessment of changes in BMD over time, which is valuable for understanding the long-term effects of cadmium exposure. The study’s primary limitations include its relatively short duration of 3 years, which may be insufficient to fully capture the long-term effects of cadmium on BMD. To address this, future research should consider extending the follow-up period to at least 10–15 years, allowing for a more comprehensive assessment of cadmium’s impact on bone health. Additionally, the sample size, particularly for subgroup analyses such as those with high U-Cd levels and male participants, may have been inadequate, potentially limiting the statistical power of these analyses. To enhance our understanding of cadmium’s effects on bone health, it is recommended that future studies incorporate a broader range of bone health biomarkers beyond BMD. Direct measurements of environmental cadmium levels during the study period were not collected. This limits the confirmation of current exposure and should be addressed in future research. Specific data on menopausal status were not obtained. Although age categories were recorded, this missing information may confound the interpretation of BMD changes in female participants.

## Conclusion

In this three-year longitudinal study of a cadmium-exposed cohort, baseline urinary cadmium was not a statistically significant predictor of the subsequent rate of change in bone mineral density. This finding contrasts with previous long-term evidence from the same population and suggests that a three-year period may be insufficient to detect the slow, cumulative skeletal effects of chronic cadmium exposure. While baseline cadmium was not predictive, other factors, including age, sex, BMI, and diabetes status, were significant determinants of short-term bone density changes. Our results underscore the critical importance of continued long-term monitoring to fully characterize the impact of environmental toxicants like cadmium on bone health.

## Supporting information

S1 Data Data. (XLSX)
